# Metagenomic Sequencing Reveals the Taxonomic and Functional Characteristics of Rumen Micro-organisms in Gayals

**DOI:** 10.3390/microorganisms11051098

**Published:** 2023-04-22

**Authors:** Huan Gao, Ye Yu, Yaqi Lv, Deao Wang, Haonan Li, Zhe Li, Yuchen Zhang, Lan Chen, Jing Leng

**Affiliations:** 1 Key Laboratory of Animal Nutrition and Feed Science of Yunnan Province, Yunnan Agricultural University, Kunming 650201, China; 2 Faculty of Animal Science and Technology, Yunnan Agricultural University, Kunming 650201, China

**Keywords:** Gayals, rumen, metagenome, carbohydrate degradation, fiber degradation mode

## Abstract

As a semi-wild breed, Gayals have a strong fiber degradation capacity, which is unique to the microbial structure and function of their rumen. In this study, the unique rumen microbial composition and function of Gayals were investigated by metagenomic sequencing, with the Yunnan yellow cattle as the control. We compared the differences in rumen micro-organisms between Gayals and the Yunnan Yellow cattle, and the results showed that there were differences in bacteria, archaea and fungi between Gayals and the Yunnan Yellow cattle, while no significant abundance changes were observed in the protozoa. In addition, the ratio of Firmicutes to Bacteroidetes (1.06) in Gayals was higher than that of the Yunnan Yellow cattle (0.66). Three enzymes (PTA, ACH and FTHFS) related to the acetate production pathway and five enzymes (BHBD, THL, PTB, BK and BCACT) involved in butyric acid production were annotated in this study. The CAZymes search results showed that the abundance of GH5, GH26, GH94, CBM11 and CBM63 in Gayals was higher than in the Yunnan Yellow cattle (*p* < 0.05). Furthermore, this research constructed a model of rumen micro-organisms degrading fibers according to the characteristics and differences in the rumen microbiota structures and functions of the two breeds. This study expands our knowledge of the rumen microbiota and the mechanisms of fiber degradation in Gayals.

## 1. Introduction

Cellulose biomass is the most plentiful and renewable resource on earth, which can provide important raw materials for industries such as papermaking, chemical industry and bioenergy [1]. Nevertheless, cellulose is refractory to degradation due to its complex structure, so its application is greatly limited. At present, the contradiction between the environmental pollution problems and the increasing energy demand is becoming more and more prominent, so the research on the efficient utilization of cellulose material resources has become increasingly essential and prevalent [2]. The rumen is one of the most important organs of ruminants and is considered to be a natural and efficient fermentation vessel for crude fiber [3]. The rumen is home to a large number of complex microbial communities. These micro-organisms degrade the lignocellulosic biomass that cannot be directly used by the host to generate monomers, which are further degraded into different microbial end-products (e.g., volatile fatty acids (VFA), hydrogen (H2), etc.) as sources of nutrients and energy for the host [4].

Fiber degradation relies mainly on the synergistic actions of multiple carbohydrate-active enzymes (CAZymes) generated by the rumen microbiota. CAZyme primarily includes glycoside hydrolases (GHs), carbohydrate esterases (CEs), carbohydrate-binding modules (CBMs), etc. Among the CAZyme family, GHs are the richest and most diverse class of enzymes that mainly play a role in hydrolyzing the glycosidic bonds of the carbohydrates in plant polysaccharides [5]. CBMs are carbohydrate-binding non-catalytic protein domains that can specifically bind cellulose and promote the degradation efficiency of cellulase [6]. Metagenomic sequencing has been demonstrated to be an effective method for investigating the rumen microbiome, both to exclude the limitations of culture conditions and to allow for a more precise microbial quantification and lineage analysis. Metagenomic analysis helps to understand the phylogenetic and metabolic characteristics of individual genomes, which are often used to study the interaction between the ruminant microbes and the organism [7,8,9].

Gayals are a semi-wild bovid species that can consume bamboo, reeds, leaves and other plant biomass. They show a strong ability to survive and have a higher mature body weight than cattle maintained in similar environments [10]. It was found that the in vitro digestibility of crude fiber dry matter, such as bamboo stem, bamboo branch, bamboo leaf, rice straw, barley straw and alfalfa in Gayals was higher than that of the Yunnan Yellow cattle [11]. Further studies found that 23.3% of the total rumen bacteria in Gayals were fiber-degrading taxa [10], which was higher than that of the local yellow cattle [12]. In addition, metaproteomic analyses showed that β-glucosidase and 6-phospho-β-glucosidase were more expressed in the rumen of Gayals than in the Yunnan Yellow cattle [13]. In summary, it was shown that Gayals have a unique rumen microbial composition, as well as cellulolytic bacteria and cellulase enzymes for efficient fiber degradation. However, there is still limited information on the rumen microbes of Gayals. Therefore, this research will investigate the rumen microbial structure and function of Gayals using metagenomic sequencing technology.

## 2. Materials and Methods

### 2.1. Ethical Statement

This study was approved by the Institutional Animal Care and Use Committee of Yunnan Agricultural University (No.: YNAU20200225).

### 2.2. Experimental Animals and Sample

Three grazing Gayals and three Yunnan Yellow cattle were selected from the same farm in Nujiang, Yunnan, China, at an altitude of 2018–2260 m from sea level. The animals had grazed freely on natural pastures and had received a supplement of salt (no supplementation of other feeds) every 2–4 weeks. All experimental animals were slaughtered, and samples of the rumen content were collected within 30 min of slaughter. All samples were immediately frozen after collection in liquid nitrogen and stored at −80 °C until DNA extraction.

### 2.3. Total DNA Extraction and Metagenomic Sequencing

Total community DNA was extracted using the E.Z.N.A.^®^ Soil DNA Kit (Omega Bio-tek, Norcross, GA, USA). DNA concentration was detected using TBS-380. Nanodrop2000 (Thermo Fisher Scientific, Waltham, MA, USA) and 1% agarose gel electrophoresis were used to check the quality and purity of the DNA. Illumina TruSeq genomic DNA libraries were constructed, and sequencing was performed on an Ilumina Hiseq 2000 platform.

### 2.4. Sequencing Data Analysis

Raw data obtained from six metagenomic samples were first filtered for reads with the adapter contamination at the end of the reads and reads <50 bp by SeqPrep (https://github.com/jstjohn/SeqPrep, accessed on 1 June 2022). Afterward, the program Sickle (https://github.com/najoshi/sickle, accessed on 1 June 2022) was utilized to remove the reads of low quality (quality score < 20) and ambiguous “N” bases (bases that cannot be determined are denoted as N). Then, further analyses of high-quality pair-end and single-end reads were mapped to the NCBI nonredundant database.

### 2.5. Metagenome Library Construction, Sequencing and Assembly

The software MEGAHIT (version 1.1.1) was used to perform the de novo assembly of the cleaning sequence. Only contigs over 300 bp were selected for further analysis. MetaGene (http://metagene.cb.k.u-tokyo.ac.jp/, accessed on 1 June 2022) was used to perform open reading frame (ORF) predictions within contigs. Then, non-redundant genes were constructed by clustering the predicted genes from all samples using CD-HIT (http://www.bioinformatics.org/cd-hit/, accessed on 1 June 2022).

### 2.6. Bioinformatics Analysis

BLASTP (BLASTVersion2.2.28+, http://blast.ncbi.nlm.nih.gov/Blast.cgi, accessed on 1 June 2022, e-value ≤ 10^−5^) was used to align the non-redundant gene set sequences with the NR database to obtain species annotation information for phyla and genera. The abundance of the taxa was calculated based on the total gene abundance relative to the taxa.

We utilized BLASTP to annotate the gene set sequences with the Kyoto Encyclopedia of Genes and Genomes (KEGG) database and the Carbohydrate-Active enZYmes (CAZy) database to analyze the gene functions of Gayals and Yunnan Yellow cattle.

### 2.7. Statistical Analysis

The differences in rumen micro-organisms between Gayals and Yunnan Yellow cattle were further analyzed using SPSS 21.0 software. Data or transformed data with a normal distribution were used to perform *t*-tests, while other data were tested using non-parametric tests [14], where each group of data was expressed as “mean ± standard deviation”.

## 3. Results

### 3.1. Metagenomic Sequences Data Statistics

After quality control, a total of 880,782,391 clean reads were generated, with 146,797,065 ± 3,134,977 reads per sample (Table 1). After the de novo assembly, an average of 534,978 contigs were obtained with an N50 of 1423 bp (range 1214–1931 bp). A total of 5,550,360 ORFs were obtained from all samples.

### 3.2. Taxonomic Composition of the Rumen Microbiota

#### 3.2.1. Taxonomic Composition of Rumen Bacteria

**Figure 1:**
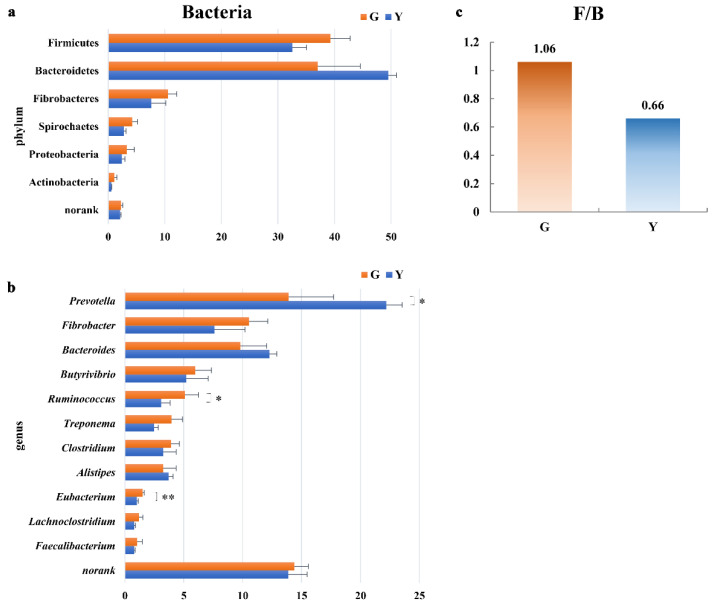
Difference analysis of bacterial phyla and genera between Gayals and Yunnan Yellow cattle. G: Gayals, Y: Yunnan Yellow cattle; (**a**,**b**): The *Y*–axis is the bacterial phylum and genus taxa, *X*–axis is the relative abundance of taxa. (**c**) The *Y*–axis is the ratio of Firmicutes to Bacteroidetes. *: *p* < 0.05, **: *p* < 0.01; the following figure is the same.

The metagenomic analysis showed that more than 92% of the taxa belonged to bacteria, which were the predominant taxa in the rumen of Gayals and the Yunnan Yellow cattle. As shown in Figure 1, at the phylum level, Firmicutes, Bacteroidetes and Fibrobacteres were the most abundant taxa in Gayals and the Yunnan Yellow cattle. Among them, the highest abundance in the rumen of Gayals was Firmicutes, which was higher than that of the Yunnan Yellow cattle (*p* > 0.05). The Bacteroidetes was the dominant phylum in the rumen of the Yunnan Yellow cattle, whose abundance was higher than that of Gayals (*p* > 0.05). The ratio of Firmicutes to Bacteroides in Gayals was 1.06, which was higher than 0.66 in the Yunnan Yellow cattle.

At the genus level, *Prevotella*, *Fibrobacter*, *Bacteroides*, *Butyrivibrio* and *Ruminococcus* were the dominant taxa in the two breeds. The relative abundance of *Prevotella* in the Yunnan Yellow cattle was higher than those in Gayals (*p* < 0.05). The relative abundance of *Ruminococcus* and *Eubacterium* in the Gayals was higher than those in the Yunnan Yellow cattle (*p* < 0.05). Moreover, the abundance of *Fibrobacter* and *Butyrivibrio* was higher in Gayals, while the abundance of *Bacteroides* was higher in the Yunnan Yellow cattle (*p* > 0.05).

#### 3.2.2. Taxonomic Composition of Rumen Fungi

**Figure 2:**
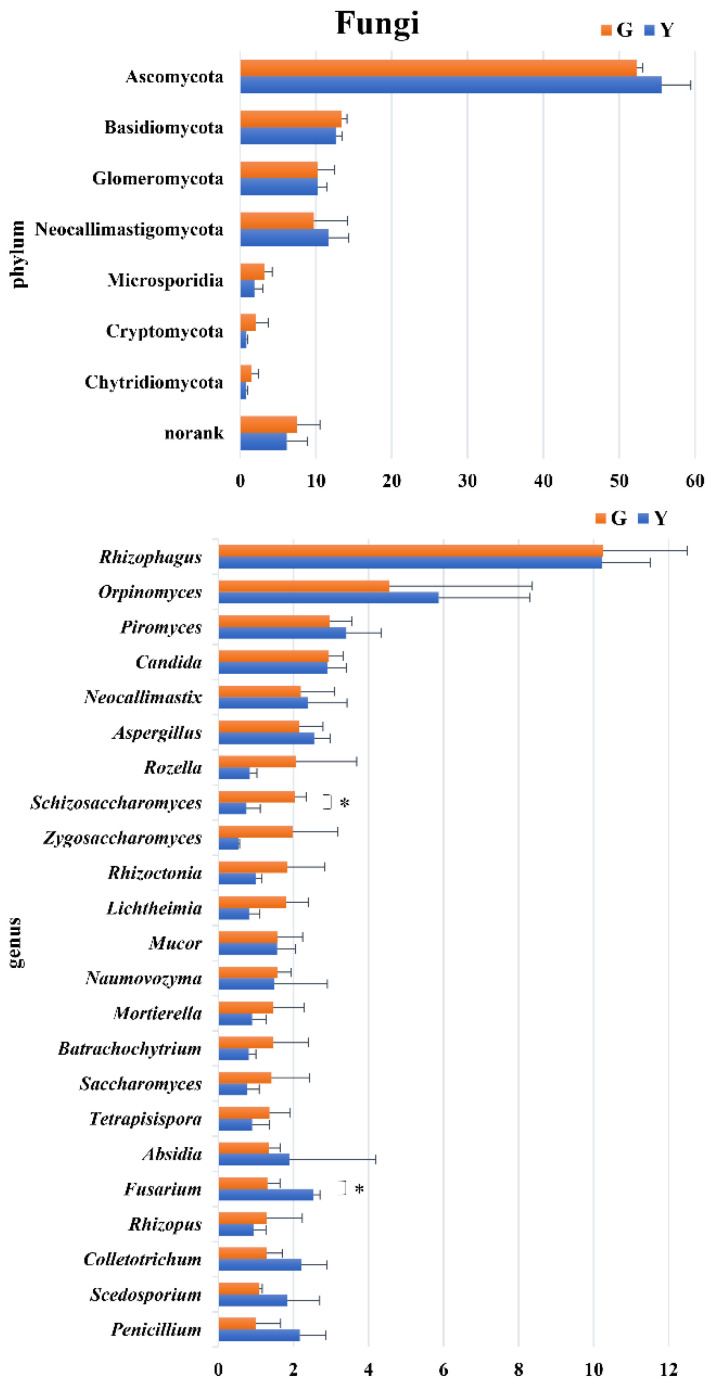
Difference analysis of fungi phyla and genera between Gayals and Yunnan yellow cattle. The *Y*–axis is the bacterial phylum and genus taxa, *X*–axis is the relative abundance of taxa. *: *p* < 0.05.

At the fungal phylum level (Figure 2), nine taxa were annotated in this study, seven of which had an abundance greater than 1%. The taxonomic analysis revealed that Ascomycota, Basidiomycota, Glomeromycota and Neocallimastigomycota were the most abundant phyla in Gayals and the Yunnan Yellow cattle.

At the genus level, compared with the Yunnan Yellow cattle, a higher abundance of *Schizosaccharomyces* and a lower abundance of *Fusarium* (*p* < 0.05) were present in the Gayals.

#### 3.2.3. Taxonomic Composition of Rumen Protozoa

**Figure 3:**
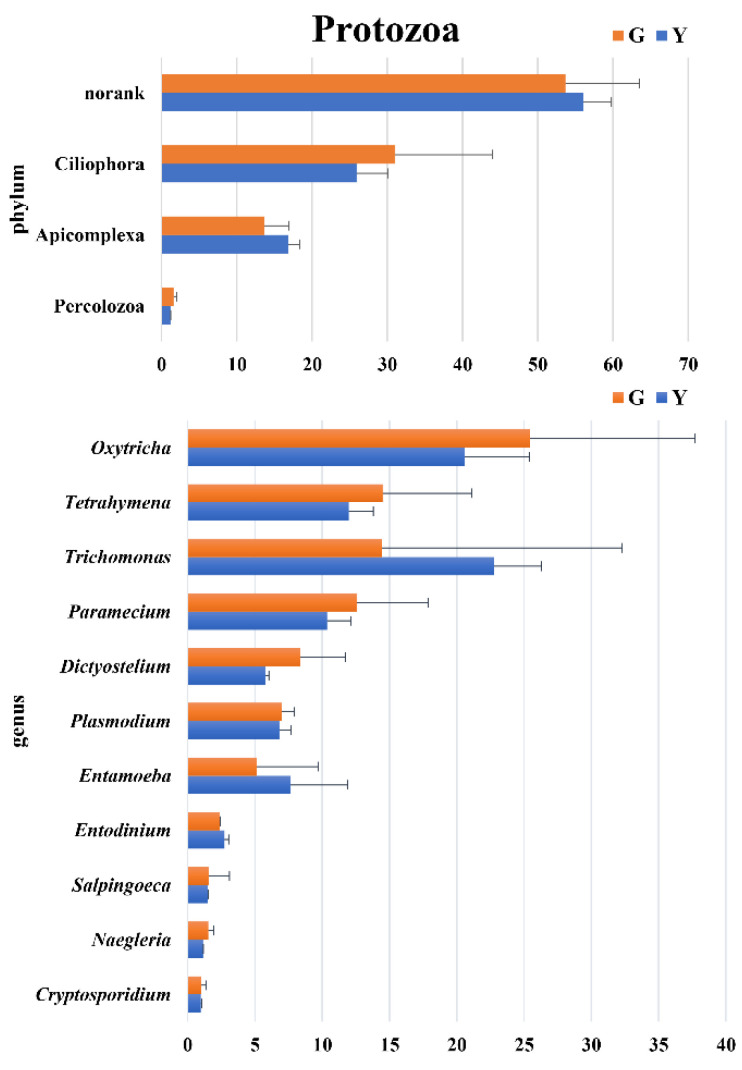
Difference analysis of protozoal phyla and genera between Gayals and Yunnan Yellow cattle. The *Y*–axis is the bacterial phylum and genus taxa, *X*–axis is the relative abundance of taxa.

As shown in Figure 3, more than 50% of the norank protozoa were present in Gayals and the Yunnan Yellow cattle. The other phyla were annotated to a total of three protozoa, Ciliophora, Apicomplexa and Percolozoa.

At the genus level, nine taxa had an abundance greater than 1%, with their abundance in Gayals being similar to those of the Yunnan Yellow cattle.

#### 3.2.4. Taxonomic Composition of Rumen Archaea

**Figure 4:**
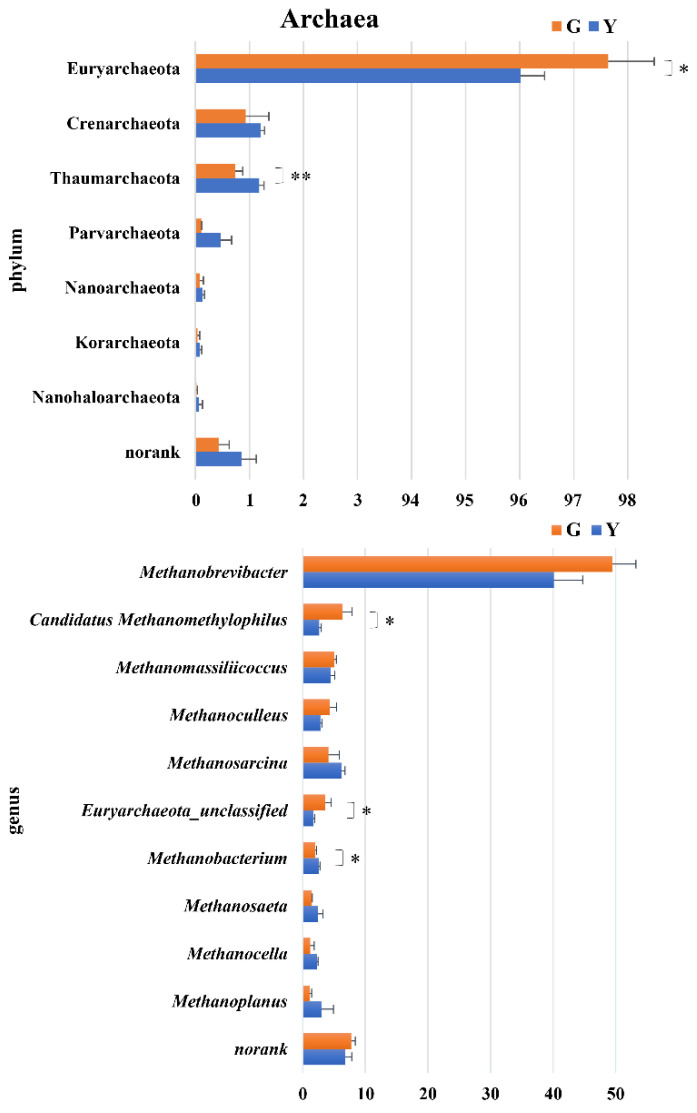
Difference analysis of archaea phyla and genera between Gayals and Yunnan Yellow cattle. The *Y*–axis is the bacterial phylum and genus taxa, *X*–axis is the relative abundance of taxa. *: *p* < 0.05, **: *p* < 0.01.

As shown in Figure 4, at the archaeal phylum level, taxonomic profiling revealed that more than 96% of the archaea belonged to Euryarchaeota in the Gayals and the Yunnan Yellow cattle. The relative abundance of Euryarchaeota in the Gayals was higher than those in the Yunnan Yellow cattle (*p* < 0.05). The abundance of Thaumarchaeota in the Gayals was lower than those in the Yunnan Yellow cattle (*p* < 0.05).

At the genus level, the abundance of *Candidatus Methanomethylophilus* and *Euryarchaeota_unclassified* in the Gayals was higher than those in the Yunnan Yellow cattle (*p* < 0.05). The relative abundance of *Methanobacterium* in the Gayals was lower than those in the Yunnan Yellow cattle (*p* < 0.05).

### 3.3. Key Enzymes and Micro-organisms Involved in Acetic Acid Production Pathway

During the carbohydrate metabolism of the rumen, complex carbohydrates are degraded into sugars by various enzymes. These sugars are used by micro-organisms to convert into pyruvic acid, which eventually produces acetic acid, propionic acid, butyric acid, methane and other products. In these metabolic pathways, acetic acid and butyric acid have a common precursor, which is acetyl coenzyme A (acetyl-CoA). The key enzymes in the metabolic pathways of acetic acid and butyric acid, with acetyl-CoA as the common metabolic precursor, were analyzed in detail in this study.

**Figure 5:**
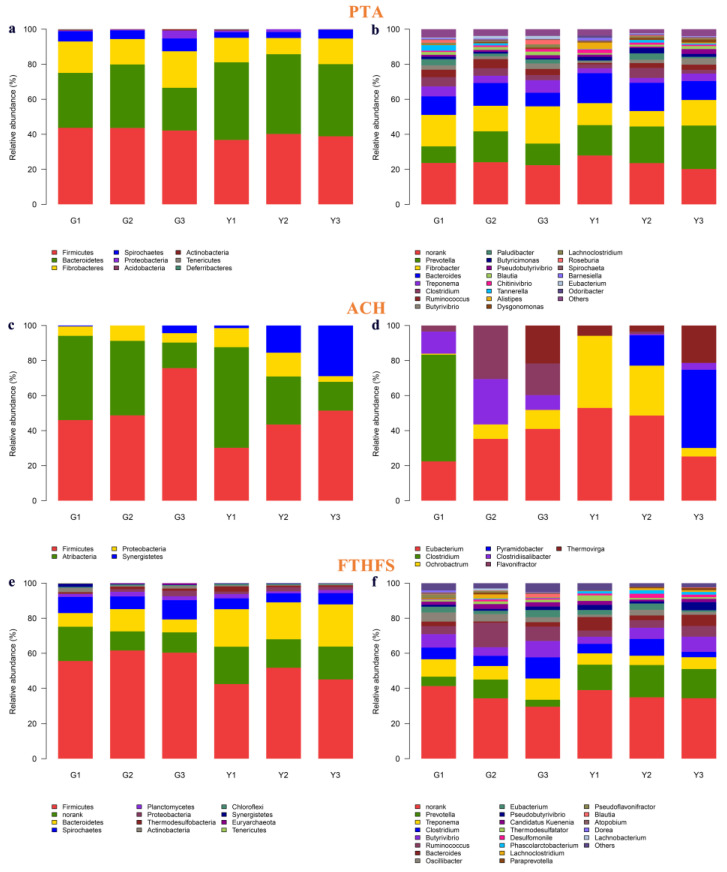
Taxonomic distribution of phyla and genera of PTA, ACH, FTHFS. (**a**,**b**): Taxonomic distribution of phyla and genera of PTA. (**c**,**d**): Taxonomic distribution of phyla and genera of ACH. (**e**,**f**): Taxonomic distribution of phyla and genera of FTHFS. The Y-axis is the relative abundance, the X-axis is the sample name. Different colors correspond to different taxa, and the length of the color block indicates the relative abundance of the taxa represented by the color.

Three key enzymes in the production of acetic acid were detected in this study, including phosphotransacetylase (PTA), acetyl-CoA hydrolase (ACH) and formyltetrhydrofolate synthetase (FTHFS). The abundance of the major micro-organisms (phylum and genus) encoding these key enzymes in the acetate production pathway is shown in Figure 5 and Table S1. The main phyla encoding PTA were Firmicutes, Bacteroidetes, Fibrobacteria and Spirochaetes. The main phyla encoding ACH were Atribacteria and Firmicutes. The main phyla encoding FTHFS were Firmicutes, Spirochaetes, Bacteroidetes, Actinobacteria, Proteobacteria and Planctomycetes. Firmicutes are involved in all pathways of acetic acid production in Gayals and the Yunnan Yellow cattle. The phylum Firmicutes, which encodes PTA and ACH, was higher in Gayals than in the Yunnan Yellow cattle. The abundance of Bacteroidetes encoding PTA and FTHFS was lower in Gayals than in the Yunnan Yellow cattle.

At the genus level, norank was the dominant taxa encoding the PTA and FTHFS pathways in Gayals and Yunnan Yellow cattle. Among the microbiota encoding the PTA pathway, *Fibrobacter*, *Treponema* and *Ruminococcus* were higher in abundance in Gayals than in the Yunnan Yellow cattle, while *Prevotella* and *Bacteroides* were in lower abundance in Gayals than in the Yunnan Yellow cattle (*p* > 0.05). The microbiota encoding FTHFS with an abundance higher than 1% were *Treponema*, *Ruminococcus*, *Clostridium*, *Butyrivibrio*, *Prevotella*, *Oscillibacter*, *Eubacterium*, *Bacteroides* and *Pseudobutyrivibrio*. Except for *Prevotella*, *Bacteroides* and *Pseudobutyrivibrio*, the other six taxa were higher in Gayals than in the Yunnan Yellow cattle. The lower abundance genera encoding the ACH in the rumen were *Clostridium*, *Eubacterium*, *Clostridiisalibacter*, *Flavonifractor*, *Thermovirga*, *Ochrobactrum* and *Pyramidobacter*.

### 3.4. Key Enzymes and Micro-organisms Involved in Butyric Acid Production Pathway

**Figure 6:**
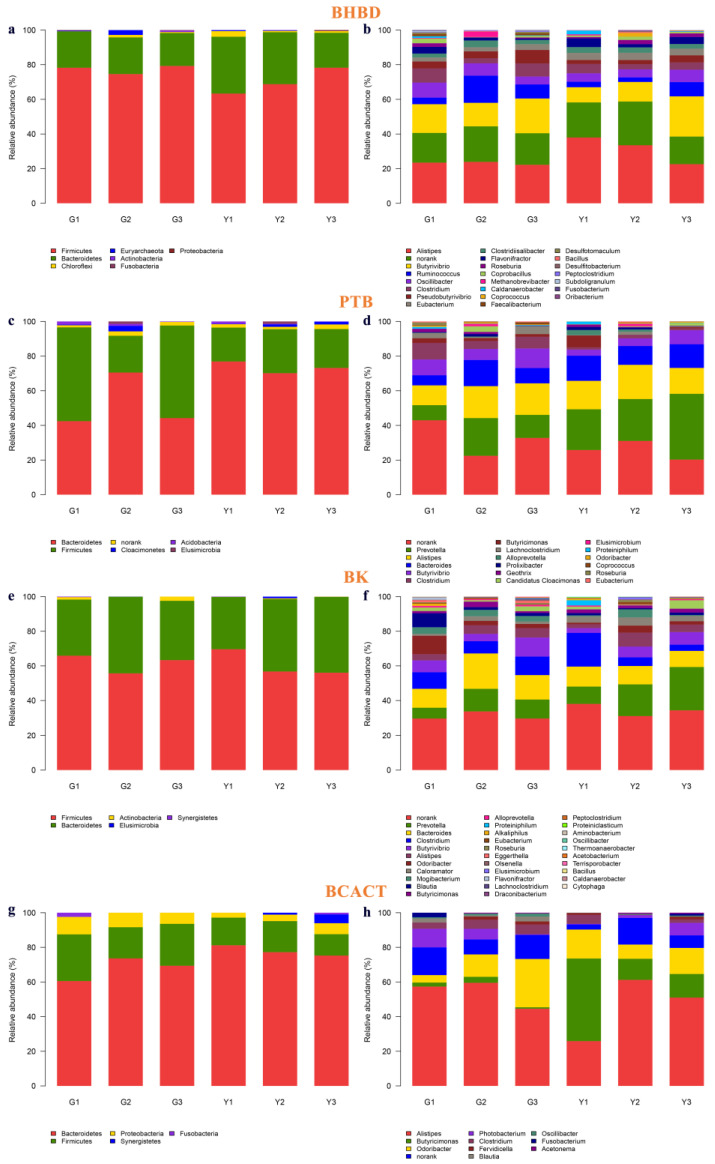
Taxonomic distribution of phyla and genera of BHBD, PTB, BK and BCACT. (**a**,**b**): Taxonomic distribution of phyla and genera of BHBD. (**c**,**d**): Taxonomic distribution of phyla and genera of PTB. (**e**,**f**): Taxonomic distribution of phyla and genera of BK. (**g**,**h**): Taxonomic distribution of phyla and genera of BCACT. The Y-axis is the relative abundance, the X-axis is the sample name. Different colors correspond to different taxa, and the length of the color block indicates the relative abundance of the taxa represented by the color.

There are two pathways for the production of butyric acid using acetyl-CoA as a substrate, one is the central pathway from acetyl-CoA to butyryl coenzyme A, and the other is the pathway from butyryl coenzyme A to butyric acid. In this study, five related enzymes were detected, which were BHBD, THL, PTB, BK and BCACT (Figure 6 and Table S2).

At the phylum level, the main phyla encoding BHBD, PTB, BK and BCACT are Firmicutes and Bacteroidetes. The phylum encoding THL was only Firmicutes. The abundance of Firmicutes (higher) and Bacteroides (lower) in Gayals was distinguished from that of the Yunnan yellow cattle.

At the genus level, the main genera encoding BHBD were *Alistipes*, *Butyrivibrio*, *Ruminococcus*, *Oscillibacter* and norank. The lower abundance of microbiota encoding the THL were *Desulfitobacterium*, *Oscillibacter*, *Clostridium*, *Acetobacterium* and *Coprococcus*, respectively. The main genera encoding BCACT were *Alistipes* and *Butyricimonas*. Norank was the most abundant taxa encoding the PTB and BK pathways in Gayals and the Yunnan Yellow cattle. Furthermore, the other main genera encoding PTB were *Alistipes*, *Prevotella*, *Bacteroides*, *Butyrivibrio* and *Clostridium*. The abundance of *Clostridium* was higher in Gayals than in the Yunnan Yellow cattle (*p* < 0.05). The other main genera encoding BK were *Bacteroides*, *Prevotella*, *Clostridium*, *Butyrivibrio*, *Odoribacter*, *Alistipes*, *Blautia*, *Mogibacterium*, *Pseudobutyrivibrio* and *Caloramator*. The abundance of *Caloramator* was lower in Gayals than in the Yunnan Yellow cattle (*p* < 0.05).

### 3.5. Enzymes and Micro-organisms Related to Fiber Degradation in Rumen

**Figure 7:**
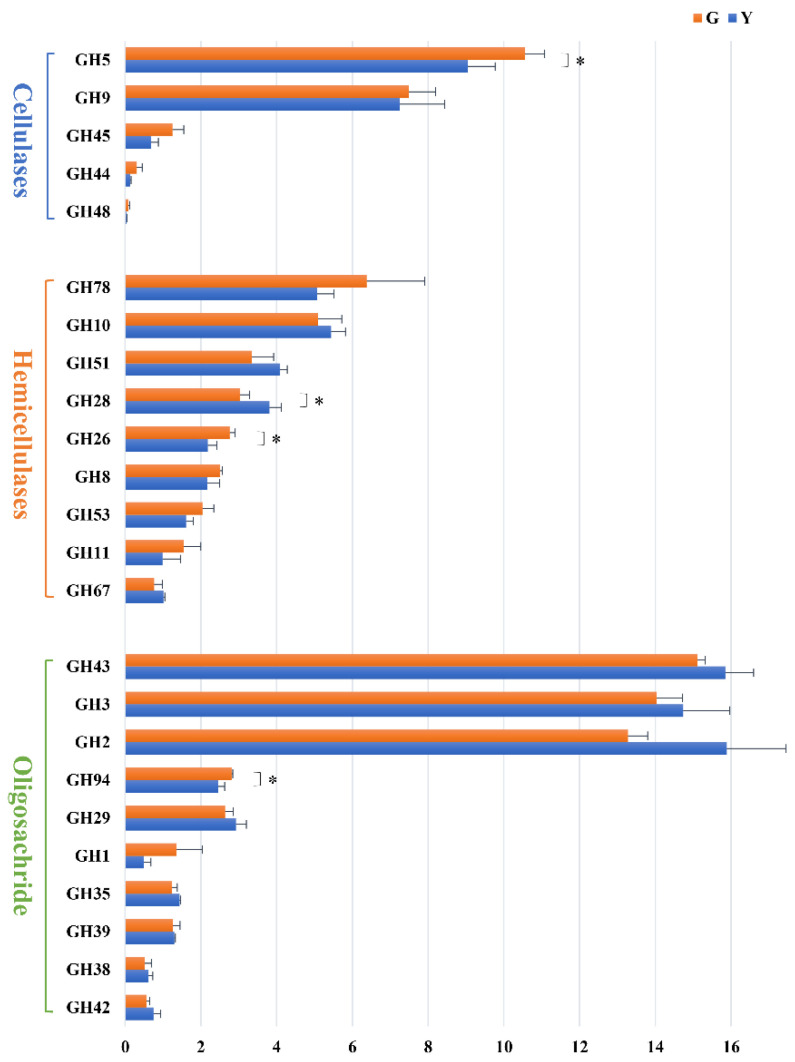
Difference analysis of GHs of fiber degradation and mean reads. *: *p* < 0.05.

The CAZymes search results showed that a total of 24 GH families were annotated to be engaged in fiber degradation (Figure 7), with transcripts encoding GHs potentially involved in cellulose(cel-GHs), hemicellulose(hemi-GHs) and oligosaccharide(oligo-GHs) degradation being 5, 9 and 10, respectively. We analyzed the taxa encoding the GH family. At the genus level, sequences encoding cel-GHs, hemi-GHs and oligo-GHs were mainly derived from 16 bacteria, 1 fungus and 2 protozoa. The five most abundant taxa encoding GH families were *Fibrobacter*, *Prevotella*, *Bacteroides*, *Ruminococcus* and *Butyrivibrio*.

Five GH families belonging to cel-GHs, i.e., GH5, GH9, GH45, GH44 and GH48, were annotated in the Gayals and the Yunnan Yellow Cattle. The most dominant cel-GHs enzyme system was GH5, which was more expressed in the Gayals than in the Yunnan Yellow cattle (*p* < 0.05), and the same trend was observed for GH45 and GH48 (*p* > 0.05). As shown in Table 2, GH45 was only found in *Fibrobacter*, and GH48 was only found in *Ruminococcus*. GH5 was found in all taxa (except *Ovis* and *Piromyces*). The abundance of *Fibrobacter* and *Ruminococcus* in the Gayals was higher than that of the Yunnan Yellow cattle (*p* > 0.05).

Nine GH families responsible for hemi-GHs, i.e., GH78, GH10, GH51, GH28, GH26, GH8, GH53, GH11 and GH67, were identified in the Gayals and the Yunnan Yellow cattle. The abundance of GH26 (higher) and GH28 (lower) in the Gayals were significantly different from the Yunnan Yellow cattle (*p* < 0.05).

Overall, 10 GH families responsible for oligo-GHs, i.e., GH43, GH3, GH2, GH94, GH29, GH1, GH35, GH39, GH38 and GH42, were identified in the Gayals and the Yunnan Yellow cattle. The abundance of GH94 in the Gayals was higher than that of the Yunnan Yellow cattle (*p* < 0.05).

**Figure 8:**
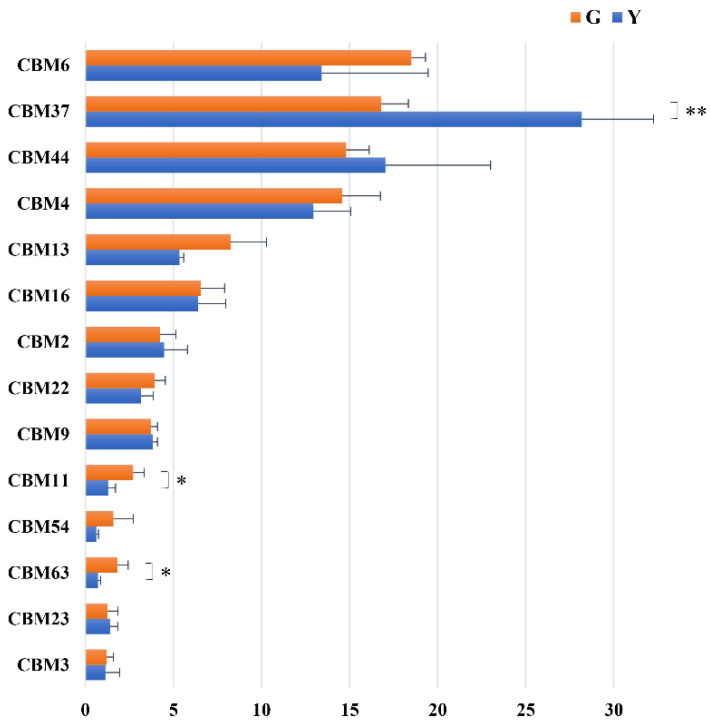
Difference analysis of CBMs of fiber degradation and mean reads. *: *p* < 0.05, **: *p* < 0.01.

Altogether, 14 CBM families were annotated to be involved in the cellulose-degrading catalytic domains (Figure 8). CBM6, CBM37, CBM44 and CBM4 were the four dominant CBM families in the rumen, with CBM6 and CBM37 being the most dominant CBM families in Gayals and the Yunnan Yellow cattle, respectively. The abundance of CBM11, CBM63 (higher) and CBM37 (lower) in the Gayals were significantly different from the Yunnan Yellow cattle (*p* < 0.05).

### 3.6. Construction of Rumen Fiber Degradation Models for Gayals and Yunnan Yellow Cattle

**Figure 9:**
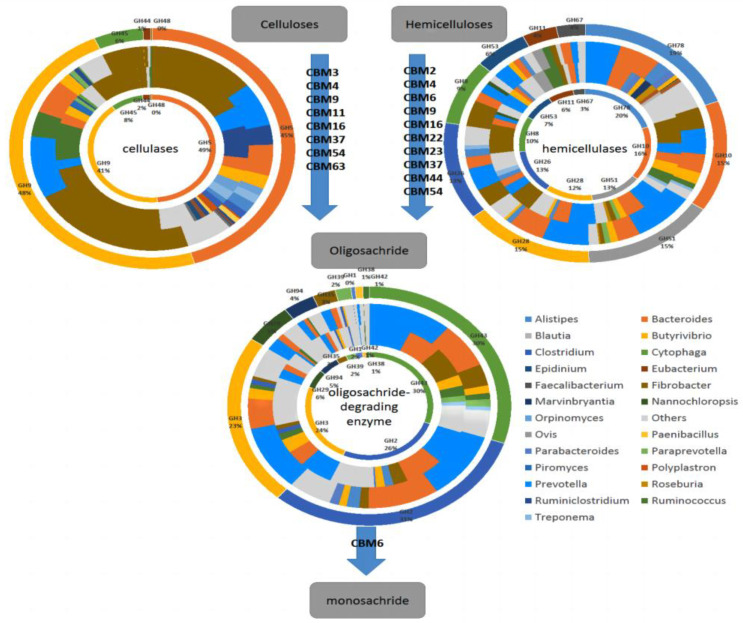
Crude fiber degradation model.

A model for microbial fiber degradation in Gayals and the Yunnan Yellow cattle was constructed by analyzing the enzymes and microbiota involved in fiber degradation (Figure 9). In this figure, the first circle from the inside to the outside is the GH family annotated by each enzyme system in the Gayals. The second circle is the species corresponding to each GH family in the Gayals. The third circle is the species corresponding to each GH family in the Yunnan Yellow cattle. The fourth circle is the GH family annotated by each enzyme line in the Yunnan Yellow cattle. The CBM families involved in fiber degradation are listed in the arrows.

The differences in these key GH families and taxa between the two breeds have been described in previous sections. In addition, CBM3, CBM4, CBM9, CBM11, CBM16, CBM37, CBM54 and CBM63 were involved in the degradation of cellulose. CBM2, CBM4, CBM6, CBM9, CBM16, CBM22, CBM23, CBM37, CBM44 and CBM54 were involved in the degradation of hemicellulose. Only CBM6 was involved in oligosaccharide degradation.

## 4. Discussion

Studies have indicated that the composition of rumen micro-organisms varies according to genetic background [8,15]. This study compared and analyzed the microbial communities and functions in the rumen of Gayals and the Yunnan Yellow cattle. Our research found that Firmicutes, Bacteroidetes and Fibrobacteres were the three dominant phyla in the rumen of Gayals and the Yunnan Yellow cattle. Bacteroides are the major phylum in the rumen [16]. In our research, the abundance of Bacteroides in the Yunnan Yellow cattle was 49.48%, which was higher than the 37.02% in the Gayals. The abundance of the Bacteroides (37.02%) was close to that of Firmicutes (39.29%) in the rumen of the Gayals. The Firmicutes to Bacteroidetes ratio (F/B) of 1.06 in the Gayals is higher than that of the Yunnan Yellow cattle (F/B = 0.66). Previous studies have found higher F/B ratios in the rumen of cows fed hay compared to those fed grain [17]. Compared to the yellow cattle and dairy cattle, higher F/B ratios were present in Yak rumen [18]. Furthermore, the F/B ratio was found to be associated with obesity in humans, presumably because the ability of Firmicutes to take in calories from food is more effective [19]. The above results suggested that the higher F/B values of Gayals partially explained their potential for efficient utilization of fibrous materials. Notably, *Prevotella* is the most dominant genus in the phylum Bacteroidota. The main role of *Prevotella* is the production of succinate and acetate from starch and protein [20]. In this study, the abundance of *Prevotella* in the Yunnan Yellow cattle was significantly higher than that of Gayals, suggesting that the starch utilization efficiency of the Yunnan Yellow cattle may be stronger. This study found that *Fibrobacter*, *Butyrivibrio*, *Ruminococcus*, *Treponema*, *Clostridium* and *Eubacterium* were the major fiber-degrading bacteria in Gayals and the Yunnan Yellow cattle, similar to other studies [21,22]. Among them, the abundance of *Fibrobacter*, *Ruminococcus*, *Treponema* and *Eubacterium* in the Gayals was higher than that of the Yunnan Yellow cattle. The results suggested that the bacterial composition of the Gayals rumen is unique, which contributes to fiber degradation in the host.

At the archaeal level, more than 96% of the archaea belonged to Euryarchaeota in this study, which is similar to the results of previous studies [9]. Previous studies have demonstrated that methanogens can synergize with other taxa to promote forage degradation [23]. For example, the rumen bacterium *Fibrobacter* sp. was shown to have positive interactions with Methanobrevibacter sp. [24]. This study found that the abundance of *Methanobrevibacter* and *Fibrobacter* in Gayals were higher than that of the Yunnan Yellow cattle. It can be seen that the stronger fiber degradation ability of the Gayals is not only related to their unique microbiota structure but also closely related to the interaction between different micro-organisms.

At the fungal level, the fungal phyla of the Gayals were similar to those of the Yunnan Yellow cattle. However, at the genus level, the higher abundance of *Schizosaccharomyces* and lower abundance of *Fusarium* were presented in the Gayals compared to the Yunnan Yellow cattle. These differences in microbial structures may lead to differences in their functions, but the exact contribution of these micro-organisms is unknown and needs to be further investigated.

In the present study, Ciliophora were the major protozoan phylum in the Gayals and the Yunnan Yellow cattle, and similar results were found in the rumen of domesticated and wild ruminants [25]. In this study, there were no significant differences in the rumen protozoa between Gayals and Yunnan Yellow cattle, which may be because of the similar growth environment of the two breeds.

Ruminal micro-organisms fermenting feed produce VFA, which is an important source of energy required by the ruminant host. We analyzed the enzymes and micro-organisms related to the metabolic pathways of acetate and butyrate. The pathways for the generation of acetate with acetyl-CoA as the substrate were detected in this study, and three major enzymes were annotated, namely PTA, ACH and FTHFS. The main phyla encoding these enzymes were Firmicutes, Bacteroidetes, Spirochaetes, etc. Among them, Firmicutes were the most abundant taxa encoding the three enzymes and were higher in Gayals than in the Yunnan Yellow cattle. In addition, the abundance of Bacteroidetes encoding PTA and FTHFS was higher in the Yunnan Yellow cattle than in the Gayals. This study found that the most abundant microbiota encoding PTA and ACH enzymes were uncultured taxa, indicating that a great number of taxa remain to be explored. At the genus level, this study demonstrated that *Fibrobacter*, *Prevotella*, *Bacteroides*, *Treponema* and *Ruminococcus* were the major taxa encoding PTA; *Clostridium*, *Eubacterium*, *Clostridiisalibacter*, *Flavonifractor and Thermovirga* were the main taxa encoding ACH; *Treponema*, *Ruminococcus*, *Clostridium*, *Butyrivibrio* and *Prevotella* were the main taxa encoding FTHFS. Among them, *Fibrobacter* (only encoding the PTA) as well as *Treponema*, *Ruminococcus*, *Clostridium* and *Eubacterium* (encoding three enzymes), had a higher abundance in Gayals. *Prevotella* and *Bacteroides* (encoding three enzymes) had a higher abundance in the Yunnan Yellow cattle. These findings suggested that the varied microbial structures may lead to differences in the acetic acid production between Gayals and the Yunnan Yellow cattle. In addition, five related enzymes were detected, along with the Acetyl-CoA as the substrate in the butyric acid production pathway in this study, namely BHBD, THL, PTB, BK and BCACT. *Butyrivibrio*, *Ruminococcus*, *Oscillibacter*, *Clostridium*, *Bacteroides* and *Prevotella* were the major butyric-acid-producing taxa. Among them, *Butyrivibrio*, *Ruminococcus*, *Oscillibacter* and *Clostridium* have a higher abundance in the Gayals, while *Bacteroides* and *Prevotella* have a higher abundance in the Yunnan Yellow cattle. These differences may partially explain the differences in the fiber degradation capacity between Gayals and Yunnan Yellow cattle, but the exact mechanisms need to be further investigated.

GHs are the main enzymes that degrade the lignocellulosic biomass in mammalian digestive tract microbiota [26,27]. In this research, 24 GH families were responsible for fiber degradation, including cellulases, hemicellulases and oligosaccharidases. Among the genes of cellulases, the abundance of GH5 and GH9 families was higher than other cellulase-encoding families, similar to previous reports [28,29,30]. The GH5 family is known to function as endoglucanases, mannanases and endo-xylanases and have exoglucanase activity [28]. In this study, a large number of genes were presumed to belong to the GH5 family, which were higher in the Gayals than in the Yunnan Yellow cattle. These results partially explain the stronger cellulose degradation capacity of the Gayals.

Among the genes encoding hemicellulases, the GH10 family has an endo-β-xylanase function, and it has a higher efficiency and better temperature and pH tolerance as well [31]. They are a great candidate for the hydrolysis of heterogeneous compounds acting on low-molecular-weight cellulose substrates [32]. Genes involved in transcribing these enzymes were also found in the metagenomic database of organisms that efficiently degrade lignocellulose in ruminants [33,34,35]. Furthermore, the GH78 family are the α-L-rhamnosidases that play a major role in cleaving the rhamnose from polysaccharides. This enzyme was present in high abundance in the microbiota of elephant feces [27] and cow rumen [36]. In this study, GH78 and GH10 were the two most abundant hemicellulase genes in the rumen of Gayals and the Yunnan Yellow cattle, which emphasizes the key role of GH10 and GH78 in ruminal hemicellulose degradation.

In this study, GH2, GH3 and GH43 were the dominant enzymes for oligosaccharide degradation in the rumen of Gayals and the Yunnan Yellow cattle, which is similar to the results of previous studies [37]. Moreover, the abundance of GH94 in oligosaccharide-degrading enzymes was higher in Gayals than that in the Yunnan Yellow cattle. The above results further emphasize the differences in fiber degradation between Gayals and the Yunnan Yellow cattle.

We associated glycoside hydrolases and the microbial genera in Gayals and the Yunnan Yellow cattle. In this study, more than 70% of GHs were encoded by five bacterial taxa, including *Fibrobacter*, *Prevotella*, *Bacteroides*, *Ruminococcus* and *Butyrivibrio*, which is similar to previous studies [28,36,38]. These micro-organisms can produce many diverse enzymes capable of degrading fiber, including cellulose, hemicellulose and oligosaccharides. Consistent with the composition of rumen micro-organisms, the abundance of rumen *Fibrobacter*, *Ruminococcus* and *Butyrivibrio* from the encoding GHs was higher in Gayals, and the abundance of *Prevotella* and *Bacteroides* was higher in Yunnan Yellow cattle. In conclusion, the unique rumen microbial reservoir of Gayals demonstrates its effective fiber degradation ability.

## 5. Conclusions

In general, this study revealed the characteristics of rumen micro-organisms in Gayals and the Yunnan Yellow cattle while retrieving the key enzymes and functional micro-organisms involved in the metabolic pathways of acetate and butyrate in the rumen, as well as analyzing the functional genes of rumen carbohydrate enzymes. Also, the differences in the rumen microbes and functional genes between the two varieties were compared. Finally, a model of fiber degradation by rumen micro-organisms in Gayals and the Yunnan Yellow cattle was constructed, which revealed the roles of various enzymes and rumen functional micro-organisms in fiber degradation to monosaccharides, highlighting the characteristics and differences in fiber degradation by rumen micro-organisms in Gayals and the Yunnan Yellow cattle. These differences reflect the uniqueness and potential advantages of rumen microbial resources in Gayals. This study provides a theoretical basis for exploring their genetic resources for efficient fibrous-material degradation with potential applications. We should emphasize that the sample size of this study was small and that large-scale sequencing efforts are needed in the future to fully understand the structural and functional uniqueness of the rumen micro-organisms of Gayals. 

## Figures and Tables

**Table 1:** Quality control data of rumen metagenomics.

Item	G1	G2	G3	Y1	Y2	Y3
Clean reads	159,215,532	144,433,815	148,517,286	150,279,539	140,500,483	137,835,736
Clean bases (bp)	23,683,460,283	21,514,126,650	22,093,344,676	22,374,123,929	20,910,006,607	20,516,301,816
Contigs	564,487	573,604	536,523	504,896	532,382	497,977
Contigs bases (bp)	632,832,801	685,122,613	615,685,117	679,612,644	773,370,470	584,110,955
N50 (bp)	1187	1317	1214	1631	1931	1263
ORFs	903,591	977,743	891,354	921,056	1,022,585	834,031

**Table 2:** Difference analysis of main functional micro-organism of fiber degradation and mean reads.

Taxon	Families (GH)	G (% ± SD)	Y (% ± SD)	*p*
*Fibrobacter*	5, 9, 44, 45, 8, 11, 10, 51, 53, 54, 2, 3, 39, 43, 94	29.58 ± 3.93	22.29 ± 5.54	0.14
*Prevotella*	5, 9, 10, 28, 51, 53, 67, 78, 8, 2, 3, 29, 35, 38, 39, 42, 43, 94	14.80 ± 3.12	26.24 ± 2.24	0.01
*Bacteroides*	5, 9, 8, 10, 28, 51, 53, 54, 67, 78, 2, 3, 29, 35, 38, 39, 42, 43, 94	11.97 ± 2.82	18.89 ± 1.34	0.02
*Ruminococcus*	5, 9, 44, 48, 8, 10, 11, 28, 51, 53, 67, 78, 1, 2, 3, 29, 35, 38, 39, 42, 43, 94	9.13 ± 3.61	4.81 ± 1.90	0.14
*Butyrivibrio*	5, 9, 8, 10, 11, 28, 51, 53, 67, 78, 1, 2, 3, 29, 35, 38, 39, 42, 43, 94	5.81 ± 1.51	4.98 ± 2.02	0.60
*Treponema*	5, 8, 9, 10, 28, 51, 53, 78, 1, 2, 3, 29, 35, 38, 39, 43, 94	2.37 ± 0.77	1.50 ± 0.36	0.15
*Roseburia*	5, 8, 10, 11, 23, 28, 51, 53, 78, 1, 2, 3, 29, 38, 39, 42, 43, 94	1.46 ± 0.73	0.40 ± 0.18	0.07
*Alistipes*	5, 9, 8, 10, 11, 25, 28, 53, 78, 2, 3, 29, 38, 39, 42, 43	2.16 ± 0.95	3.52 ± 0.86	0.14
*Ovis*	11	1.29 ± 0.20	0.89 ± 0.40	0.19
*Clostridium*	5, 9, 8, 10, 11, 28, 51, 53, 78, 1, 2, 3, 29, 35, 38, 39, 42, 43, 94	1.43 ± 0.60	1.29 ± 0.63	0.79
*Faecalibacterium*	5, 8, 10, 28, 51, 67, 78, 1, 2, 3, 39, 43, 94	0.96 ± 0.35	0.70 ± 0.19	0.32
*Polyplastron*	5, 10, 11	0.63 ± 0.57	0.12 ± 0.04	0.27
*Cytophaga*	5, 9, 8, 10, 67, 2, 3, 29, 43	0.70 ± 0.41	0.29 ± 0.36	0.25
*Marvinbryantia*	5, 78, 1, 2, 3, 35, 39, 43, 94	0.86 ± 0.49	0.10 ± 0.06	0.048
*Piromyces*	3, 9, 11	0.57 ± 0.51	0.06 ± 0.03	0.46
*Epidinium*	5, 10	0.46 ± 0.48	0.04 ± 0.03	0.61
*Parabacteroides*	5, 8, 10, 28, 51, 78, 2, 3, 38, 42, 43	1.03 ± 0.11	1.11 ± 0.43	0.76
*Paenibacillus*	5, 9, 8, 10, 28, 51, 53, 67, 78, 1, 2, 3, 29, 35, 38, 39, 42, 43, 94	0.90 ± 0.43	0.67 ± 0.32	0.49
*Paraprevotella*	5, 9, 10, 51, 67, 78, 2, 29, 35, 43	1.00 ± 0.20	1.14 ± 0.66	0.74
others		12.87 ± 0.89	10.96 ± 2.90	0.38

## Data Availability

The datasets generated during and/or analyzed during the current study are not publicly available due to the protection of privacy and intellectual property, but are available from the corresponding author upon reasonable request.

## Supplementary Materials

The following supporting information can be downloaded at: https://www.mdpi.com/article/10.3390/microorganisms11051098/s1, Table S1: Taxonomic distribution of phyla and genera of acetate metabolism and mean reads; Table S2: Taxonomic distribution of phyla and genera of butyric acid metabolism and mean reads.
