# Supplementary material for: Metagenomic Sequencing Reveals the Taxonomic and Functional Characteristics of Rumen Micro-organisms in Gayals
Source: Microorganisms. 2023 Apr 22;11(5):1098. doi: 10.3390/microorganisms11051098 (PMC10223140; doi:10.3390/microorganisms11051098)
Supplement: Supplementary file 1 [file microorganisms-11-01098-s001.zip › microorganisms-1944956-supplementary.pdf]

Table S1. Taxonomic distribution of phyla and genera of acetate metabolism and mean reads.

item	taxa	G(%±SD)	Y(%±SD)	*p* value
phyla				
PTA	Firmicutes	23.39±1.01	21.39±0.23	0.07
PTA	Bacteroidetes	16.67±3.59	24.23±1.35	0.03
PTA	Fibrobacteres	9.60±1.51	7.02±1.80	0.13
PTA	Spirochaetes	3.26±0.56	2.17±0.55	0.04
PTA	Acidobacteria	0.04±0.02	0.18±0.24	0.43
PTA	Actinobacteria	0.23±0.14	0.02±0.01	0.04
PTA	Deferribacteres	0.00±0.00	0.02±0.04	0.51
PTA	Proteobacteria	0.94±1.24	0.45±0.38	0.58
PTA	Tenericutes	0.06±0.11	0.00±0.00	0.51
ACH	Firmicutes	1.72±0.45	0.71±0.13	0.02
ACH	Atribacteria	1.12±0.74	0.60±0.41	0.35
ACH	Proteobacteria	0.19±0.04	0.17±0.10	0.68
ACH	Synergistetes	0.05±0.07	0.25±0.21	0.18
FTHFS	Firmicutes	25.31±2.02	19.92±2.92	0.06
FTHFS	norank	5.95±1.88	8.01±0.73	0.15
FTHFS	Bacteroidetes	3.96±1.23	9.48±0.84	0.00
FTHFS	Spirochaetes	3.93±0.91	2.54±0.24	0.06
FTHFS	Actinobacteria	0.62±0.39	0.19±0.03	0.08
FTHFS	Planctomycetes	0.92±0.21	0.74±0.24	0.38
FTHFS	Proteobacteria	0.73±0.63	0.81±0.25	0.84
FTHFS	Thermodesulfobacteria	0.50±0.17	0.67±0.48	0.59
FTHFS	Synergistetes	0.33±0.38	0.11±0.05	0.41
FTHFS	Chloroflexi	0.24±0.04	0.29±0.06	0.37
FTHFS	Euryarchaeota	0.21±0.18	0.03±0.01	0.10
FTHFS	Tenericutes	0.03±0.05	0.00±0.00	0.51
genera				
PTA	*norank*	11.86±0.88	12.54±2.48	0.68
PTA	*Fibrobacter*	9.07±1.42	6.31±1.73	0.10
PTA	*Prevotella*	6.68±2.16	10.98±1.82	0.06
PTA	*Bacteroides*	5.34±1.48	7.70±1.89	0.16
PTA	*Treponema*	2.85±0.68	1.70±0.47	0.07
PTA	*Ruminococcus*	2.22±0.52	1.12±0.59	0.07
PTA	*Clostridium*	2.12±0.66	1.74±0.96	0.61
PTA	*Paludibacter*	1.25±0.52	0.81±0.86	0.49
PTA	*Butyrivibrio*	1.21±0.36	1.13±0.69	0.87
PTA	*Blautia*	0.90±0.08	0.63±0.23	0.13
PTA	*Tannerella*	0.76±0.83	0.40±0.32	0.53
PTA	*Roseburia*	0.67±0.60	0.08±0.09	0.23
PTA	*Pseudobutyrivibrio*	0.65±0.20	0.86±0.54	0.56
PTA	*Eubacterium*	0.60±0.34	0.08±0.03	0.04
PTA	*Butyricimonas*	0.53±0.22	1.17±0.31	0.04
PTA	*Lachnoclostridium*	0.51±0.35	0.28±0.17	0.37
PTA	*Chitinivibrio*	0.51±0.15	0.72±0.38	0.41
PTA	*Spirochaeta*	0.36±0.19	0.29±0.02	0.78
PTA	*Porphyromonas*	0.33±0.15	0.00±0.00	0.07
PTA	*Dysgonomonas*	0.31±0.17	0.62±0.34	0.23
PTA	*Prolixibacter*	0.22±0.11	0.30±0.16	0.53
PTA	*Barnesiella*	0.18±0.13	0.39±0.28	0.32

PTA	*Flavonifractor*	0.16±0.09	0.20±0.13	0.67
PTA	*Caldanaerobius*	0.12±0.04	0.13±0.11	0.98
PTA	*Acetobacter*	0.12±0.14	0.00±0.00	0.20
PTA	*Rikenella*	0.12±0.11	0.11±0.07	0.89
PTA	*Azospirillum*	0.10±0.17	0.00±0.00	0.51
PTA	*Oribacterium*	0.10±0.02	0.03±0.03	0.04
PTA	*Syntrophothermus*	0.06±0.08	0.15±0.25	0.67
PTA	*Alistipes*	0.05±0.07	0.79±1.12	0.29
PTA	*Holophaga*	0.04±0.02	0.18±0.24	0.70
PTA	*Odoribacter*	0.02±0.03	0.27±0.42	0.22
PTA	*Caldicellulosiruptor*	0.01±0.01	0.14±0.14	0.12
ACH	*Clostridium*	0.24±0.42	0.00±0.00	0.51
ACH	*Eubacterium*	0.23±0.04	0.22±0.11	0.94
ACH	*Clostridiisalibacter*	0.11±0.06	0.01±0.01	0.08
ACH	*Flavonifractor*	0.10±0.07	0.01	0.05
ACH	*Ochrobactrum*	0.04±0.03	0.12±0.09	0.19
ACH	*Pyramidobacter*	0.00±0.00	0.13±0.14	0.20
FTHFS	*norank*	13.82±2.00	14.74±0.47	0.48
FTHFS	*Treponema*	3.93±0.90	2.52±0.26	0.06
FTHFS	*Ruminococcus*	3.53±1.93	1.90±0.57	0.23
FTHFS	*Clostridium*	3.26±1.40	2.50±1.44	0.55
FTHFS	*Butyrivibrio*	2.89±0.90	2.63±1.02	0.75
FTHFS	*Prevotella*	2.66±1.44	6.80±1.12	0.02
FTHFS	*Oscillibacter*	1.60±0.43	0.86±0.46	0.11
FTHFS	*Eubacterium*	1.23±0.45	0.99±0.58	0.59
FTHFS	*Candidatus Kuenenia*	0.92±0.21	0.70±0.23	0.28
FTHFS	*Bacteroides*	0.79±0.42	2.28±0.97	0.07
FTHFS	*Pseudobutyrivibrio*	0.56±0.22	1.19±0.70	0.21
FTHFS	*Thermodesulfatator*	0.50±0.17	0.67±0.48	0.59
FTHFS	*Pseudoflavonifractor*	0.41±0.37	0.02±0.02	0.07
FTHFS	*Lachnoclostridium*	0.41±0.49	0.30±0.22	0.75
FTHFS	*Desulfomonile*	0.29±0.17	0.68±0.31	0.13
FTHFS	*Blautia*	0.28±0.37	0.04±0.07	0.22
FTHFS	*Atopobium*	0.25±0.22	0.01±0.01	0.35
FTHFS	*Dehalococcoides*	0.24±0.04	0.29±0.06	0.37
FTHFS	*Paraprevotella*	0.19±0.21	0.32±0.26	0.53
FTHFS	*Dorea*	0.16±0.20	0.01±0.00	0.35
FTHFS	*Thermosediminibacter*	0.16±0.15	0.03±0.04	0.14
FTHFS	*Cloacibacillus*	0.15±0.15	0.11±0.05	0.65
FTHFS	*Lachnobacterium*	0.15±0.25	0.00±0.00	0.20
FTHFS	*Bacillus*	0.14±0.18	0.01±0.01	0.11
FTHFS	*Selenomonas*	0.14±0.14	0.20±0.14	0.91
FTHFS	*Coprobacillus*	0.13±0.05	0.24±0.10	0.18
FTHFS	*Phascolarctobacterium*	0.13±0.05	0.67±0.18	0.01
FTHFS	*Azospirillum*	0.13±0.22	0.00±0.00	0.51

Table S2. Taxonomic distribution of phyla and genera of butyric acid metabolism and mean reads.

item	taxa	G(%±SD)	Y(%±SD)	*p* value
phyla				
BHBD	Firmicutes	14.2±1.37	13.45±2.08	0.63
BHBD	Bacteroidetes	3.73±0.26	5.28±1.24	0.10
BHBD	Chloroflexi	0.13±0.13	0.31±0.23	0.31
BHBD	Euryarchaeota	0.19±0.29	0.06±0.04	1.00
THL	Firmicutes	1.22±1.05	0.38±0.37	0.26
PTB	Bacteroidetes	14.8±3.38	20.41±0.87	0.17
PTB	Firmicutes	12.51±5.97	6.32±1.37	0.09
PTB	norank	0.55±0.17	0.54±0.17	0.97
PTB	Cloacimonetes	0.32±0.45	0.31±0.23	0.97
PTB	Acidobacteria	0.31±0.24	0.16±0.17	0.45
PTB	Elusimicrobia	0.12±0.21	0.14±0.24	1.00
BK	Firmicutes	28.44±1.91	28.26±5.49	0.96
BK	Bacteroidetes	17.19±3.85	17.76±3.14	0.85
BK	Actinobacteria	0.56±0.52	0.08±0.09	0.19
BK	Elusimicrobia	0.01±0.02	0.13±0.23	1.00
BCACT	Bacteroidetes	3.78±0.38	4.94±1.51	0.26
BCACT	Firmicutes	1.28±0.25	1.00±0.41	0.38
BCACT	Fusobacteria	0.46±0.11	0.25±0.03	1.00
BCACT	Synergistetes	0.00±0.00	0.10±0.11	0.20
genera				
BHBD	*Alistipes*	3.72±0.23	5.28±1.23	0.10
BHBD	*norank*	3.00±0.43	3.50±1.05	0.49
BHBD	*Butyrivibrio*	2.69±0.62	2.47±1.36	0.81
BHBD	*Ruminococcus*	1.50±1.02	0.79±0.53	0.35
BHBD	*Oscillibacter*	1.08±0.26	0.94±0.26	0.55
BHBD	*Clostridium*	0.99±0.44	0.70±0.13	0.34
BHBD	*Pseudobutyrivibrio*	0.85±0.40	0.50±0.19	0.24
BHBD	*Clostridiisalibacter*	0.46±0.16	0.52±0.06	0.62
BHBD	*Eubacterium*	0.43±0.12	0.69±0.08	0.04
BHBD	*Flavonifractor*	0.33±0.22	0.60±0.23	0.21
BHBD	*Coprobacillus*	0.20±0.19	0.21±0.12	0.93
BHBD	*Methanobrevibacter*	0.19±0.29	0.04±0.05	0.52
BHBD	*Roseburia*	0.14±0.15	0.30±0.17	0.29
BHBD	*Faecalibacterium*	0.13±0.12	0.00±0.00	0.20
BHBD	*Caldanaerobacter*	0.06±0.07	0.13±0.18	0.58
BHBD	*Desulfitobacterium*	0.01±0.01	0.14±0.22	0.36
THL	*Desulfitobacterium*	0.67±0.91	0.20±0.12	0.19
THL	*Oscillibacter*	0.20±0.23	0.02±0.02	0.59
THL	*Clostridium*	0.10±0.08	0.01±0.00	0.08
THL	*Acetobacterium*	0.08±0.06	0.03±0.05	0.30
THL	*Coprococcus*	0.04±0.04	0.05±0.08	0.99
PTB	*norank*	9.15±3.29	7.17±1.93	0.42
PTB	*Alistipes*	4.40±0.96	4.75±1.03	0.69
PTB	*Prevotella*	3.98±1.59	7.91±2.26	0.07
PTB	*Bacteroides*	2.69±1.15	3.58±0.27	0.26
PTB	*Butyrivibrio*	2.49±0.76	1.48±0.68	0.16
PTB	*Clostridium*	1.95±0.78	0.42±0.18	0.03
PTB	*Lachnoclostridium*	0.74±0.57	0.23±0.24	0.23

PTB	*Butyricimonas*	0.55±0.20	0.66±0.96	0.85
PTB	*Geothrix*	0.31±0.25	0.16±0.17	0.45
PTB	*Candidatus Cloacimonas*	0.29±0.47	0.19±0.17	0.97
PTB	*Coprococcus*	0.19±0.14	0.03±0.03	0.09
PTB	*Prolixibacter*	0.17±0.19	0.28±0.21	0.55
PTB	*Roseburia*	0.16±0.07	0.13±0.09	0.35
PTB	*Odoribacter*	0.16±0.27	0.01±0.00	0.61
PTB	*Proteiniphilum*	0.14±0.14	0.15±0.2	0.98
PTB	*Elusimicrobium*	0.12±0.21	0.14±0.24	1.00
PTB	*Alloprevotella*	0.08±0.02	0.39±0.38	0.24
BK	*norank*	13.85±1.97	15.60±2.88	0.43
BK	*Bacteroides*	6.83±2.59	4.72±0.69	0.23
BK	*Prevotella*	4.53±1.74	7.97±3.52	0.20
BK	*Clostridium*	4.03±0.58	4.33±4.30	0.91
BK	*Butyrivibrio*	3.22±1.29	2.41±1.06	0.45
BK	*Odoribacter*	2.31±2.09	1.00±0.59	0.33
BK	*Alistipes*	2.08±0.44	2.11±1.09	0.96
BK	*Blautia*	1.68±1.64	0.54±0.22	0.20
BK	*Mogibacterium*	1.60±0.23	0.80±0.94	0.27
BK	*Butyricimonas*	0.84±0.61	0.89±0.23	0.91
BK	*Pseudobutyrivibrio*	0.83±0.40	1.07±1.11	0.74
BK	*Caloramator*	0.77±0.43	1.69±0.23	0.03
BK	*Alloprevotella*	0.35±0.04	0.23±0.12	0.17
BK	*Eggerthella*	0.35±0.30	0.01±0.00	0.35
BK	*Alkaliphilus*	0.23±0.31	0.22±0.26	0.98
BK	*Eubacterium*	0.21±0.08	0.18±0.28	0.87
BK	*Flavonifractor*	0.16±0.28	0.00±0.00	0.51
BK	*Roseburia*	0.16±0.11	0.25±0.22	0.55
BK	*Olsenella*	0.15±0.15	0.07±0.09	0.49
BK	*Lachnoclostridium*	0.11±0.05	0.05±0.06	0.25
BK	*Elusimicrobium*	0.01±0.02	0.13±0.23	1.00
BK	*Proteiniphilum*	0.01±0.01	0.48±0.83	1.00
BCACT	*Alistipes*	2.83±0.53	2.59±1.36	0.79
BCACT	*Odoribacter*	0.78±0.60	0.74±0.32	0.93
BCACT	*norank*	0.67±0.18	0.50±0.48	0.61
BCACT	*Photobacterium*	0.30±0.27	0.13±0.13	0.37
BCACT	*Clostridium*	0.25±0.06	0.15±0.17	0.37
BCACT	*Butyricimonas*	0.12±0.08	1.50±1.45	0.07
BCACT	*Blautia*	0.10±0.06	0.00±0.00	0.04
